# Mental Health Trajectories in Medical Students: The Impact of Academic Repetition on Depressive Symptoms and Self-Rated Health

**DOI:** 10.3390/jcm14238447

**Published:** 2025-11-28

**Authors:** Andrea Horváth-Sarródi, Károly Berényi, Boróka Gács, Gellért Gerencsér, Boglárka Bernadett Tisza, Éva Pozsgai, István Kiss

**Affiliations:** 1 Department of Public Health Medicine, University of Pécs Medical School, Szigeti Street 12, 7624 Pécs, Hungary; andrea.sarrodi@aok.pte.hu (A.H.-S.);; 2 Department of Behavioural Sciences, University of Pécs Medical School, Szigeti Street 12, 7624 Pécs, Hungary; 3 Preclinical Research Center, Medical School, University of Pécs, Szigeti Street 12, 7624 Pécs, Hungary; 4 Doctoral School of Health Sciences, Faculty of Health Sciences, University of Pécs, Szepesy Ignác Street 1-3, 7621 Pécs, Hungary; 5 Department of Primary Health Care, University of Pécs Medical School, Rákóczi Street 2, 7623 Pécs, Hungary

**Keywords:** depressive symptoms, self-rated health, depression, medical students, academic repetition, repeaters, financial hardship, physical activity, risk factors, protective factors

## Abstract

**Background**: Medical students are at high risk for depressive symptoms, but few longitudinal studies have examined changes over time, especially among those repeating academic years. The aim of this study was to assess the prevalence and temporal trends of depressive symptoms and self-rated health among Hungarian medical students, examining the influence of demographic and lifestyle factors and the independent impact of academic repetition on mental health risk. **Methods**: In a four-year cohort study at the University of Pécs Medical School, students were classified as original-entry cohort (progressing according to the standard curriculum) or repeaters (those who began studies before 2016). Sample size was N = 196 in the first semester of the first year, N = 192 in the second semester of the first year, N = 157 in the second year, N = 147 in the third year, and N = 155 in the fourth year. Data were collected at five time points on lifestyle factors and depressive symptoms using the Beck Depression Inventory and a self-rated health questionnaire. Multivariate logistic regression identified risk and protective factors. **Results**: In the original-entry cohort, depressive symptoms peaked in the first two years (up to 52.6%), then declined to 24.6% by year four. Compared to age-matched peers, students had 3.1-fold higher odds of depressive symptoms at entry (OR = 3.10, CI: 2.007–4.773; *p* < 0.001), rising to 5.2-fold after the first exam period (OR = 5.20, CI: 3.375–8.000; *p* < 0.001). Among repeaters, odds of depressive symptoms remained over threefold higher than the general population (OR = 3.64, CI: 1.888–7.022, year IV/1), and self-rated health was significantly lower (*p* < 0.001). Financial hardship increased risk, while regular physical activity was protective. **Conclusions**: This study highlights the elevated and persistent mental health risks among repeaters and medical students with financial difficulties. Stratifying by academic progression reveals unique vulnerabilities, underscoring the need for targeted support in medical training.

## 1. Introduction

Medical students face disproportionately high levels of depressive symptoms and other mental health problems during their studies compared to their age-matched peers [1,2,3,4]. Multiple early studies already demonstrated this gap, driven by academic overload, frequent examinations, high performance expectations, early exposure to illness, and reduced opportunities for rest and social activities (Palicka et al. [5]). For example, Sherry et al. reported that first-year female medical students had significantly higher depressive mood scores (40.1 ± 8.5) than women aged 20–29 in the general population (34.4 ± 6.8) [6]. Similarly, in a sample of 304 students, Zoccolillo et al. found a lifetime prevalence of major depressive disorder at least three times higher than in the general population aged 18–24 [7]. Additional findings reinforce this pattern: Guthrie et al. observed that 36% of first-year students scored above the General Health Questionnaire (GHQ) cut-off for psychological distress [2], while Rosal et al. reported depressive symptoms in 18% of students before matriculation, rising to 39% in the second year and declining to 31% in the fourth (*p* = 0.0001) [3]. Tyssen et al. documented a one-year prevalence of suicidal ideation of 14% among Norwegian medical students, compared to the population norm of 2.3% measured with the same scale [4].

International longitudinal studies provide further evidence of increasing psychological distress over time [8,9,10]. Moffat et al. found that GHQ-12 caseness increased more than threefold from trimester 1 to trimester 3 (OR = 3.26; 95% CI: 2.25–4.72; *p* < 0.0001) [8]. Aktekin et al. observed that GHQ-12 scores among medical students increased from 9.3 to 14.3 (*p* < 0.001), and BDI scores from 6.9 to 11.1 (*p* < 0.001) between years 1 and 2, with smaller or inconsistent changes in economics and physical-education students [9]. Follow-up work by Guthrie et al. showed that GHQ caseness declined from 36.6% in year 1 to 21.9% in year 5, indicating some adaptation over the course of training [10].

Depressive symptoms among medical students have been linked to impaired academic performance, reduced empathy, heightened dropout risk, and future professional burnout [11].

A comprehensive meta-analysis of 167 cross-sectional studies across 43 countries reported a pooled prevalence of 27.2% for depression among medical students, with only approximately 16% seeking professional mental health support, while data extracted from 24 cross-sectional studies from 15 countries found the overall pooled crude prevalence of suicidal ideation to be as high as 11.1% [12]. Another systematic review of over 62,000 medical students showed a global depression prevalence of 28.0%, with first-year students experiencing the highest rates (~33.5%) and a gradual decline in later years (*p* = 0.005) [13]. 

In Hungary, nearly 20% of medical students exceeded the GHQ-12 threshold for mental health problems, and a low sense of coherence correlated with poorer psychological well-being [14]. Another cross-sectional survey at Semmelweis University identified nationality-related differences in mental health scores, showing that fourth-year Hungarian students reported lower mental well-being compared to peers from other countries studying at the same Medical School [15]. Cultural variations are known to influence mental well-being, as shown in a 12-country study that identified substantial cross-national differences in distress, burnout, and substance use among medical students [16].

Lifestyle factors, particularly physical activity, appear protective: regular leisure-time exercise has been associated with reduced depression, anxiety, and improved self-rated mental health [15,17,18]. Financial hardship and living arrangements also influence mental health, although longitudinal studies examining these relationships remain scarce, especially in Central and Eastern Europe [11,19,20].

Depressive symptoms carry important academic and professional consequences, and have been linked to poorer academic performance, reduced empathy, higher dropout risk, and later burnout [11,21,22]. Stigma may further hinder help-seeking: in one study of Indian medical students with depressive symptoms, 56.1% believed their peers would respect them less if aware of their condition (*p* < 0.001) [23]. Self-rated health (SRH) is a widely used subjective indicator that reliably reflects mental and physical well-being [24,25]. In a large Franco-Japanese study involving 5655 French and 17,148 Japanese university students, high depressive symptoms (measured with the PHQ-2) were strongly associated with poor SRH even after adjusting for sex, age, year of study, sleep quality, BMI, physical activity, smoking, and alcohol use: odds ratio (OR) = 2.82 (CI: 1.99–4.01) in France and OR = 7.10 (CI: 5.76–8.74) in Japan [26]. Participants included students from multiple institutions, highlighting the generalizability of the association between depressive symptoms and poor SRH across diverse cultural contexts. These findings highlight the value of SRH as a simple indicator of mental health status.

Despite extensive research on medical student mental health, the impact of academic repetition is poorly understood. Repeaters may face specific stressors—academic failure, peer discontinuity, and financial strain—that increase vulnerability to depression and poorer self-rated health [5,27]. In the Czech Republic, a national sample of more than 3000 medical students showed that excessive stress markedly increased the odds of voluntary dropout (OR = 4.20; CI: 3.39–5.19; *p* < 0.001). High stress was also associated with frequent somatic complaints (70%; OR = 7.97; *p* < 0.001), alcohol use for stress reduction (29.7%; OR = 2.69; *p* < 0.001), and medication use (17.9%; OR = 9.16; *p* < 0.001). Nearly half of highly stressed students contemplated discontinuing their studies (48.4% vs. 18.5%; OR = 3.91; *p* < 0.001) [5]. In an international multicenter study including 1262 medical students from Poland, Germany, and Portugal, depressive symptoms (BDI ≥ 10) were common, with the highest prevalence in Poland (56.3%), followed by Germany (34.9%) and Portugal (26.0%) [27]. Mean BDI scores likewise differed (Poland: 13.76 ± 9.99; Germany: 8.49 ± 7.64; Portugal: 7.37 ± 7.67). However, to our knowledge, few longitudinal investigations explicitly stratify outcomes by repeater status, and to date, no study in the Central European region has followed the mental health of medical students by repeater versus non-repeater groups.

In view of these gaps, we conducted a four-year cohort study to investigate the occurrence of depressive symptoms and self-rated health among medical students in a regional Hungarian medical school. Our objectives were to assess the prevalence and temporal changes in these outcomes within the cohort, to examine the influence of demographic and lifestyle variables—such as age, gender, financial circumstances, living arrangements, and physical activity—and to determine whether academic repetition independently contributes to mental health risk.

## 2. Methods

### 2.1. Study Setting

The study was conducted at the University of Pécs Medical School, one of only four medical faculties in Hungary offering a six-year graduate medical program. The institution enrolls a diverse student population and follows a standardized curriculum consistent with national medical education guidelines. Enrollment in the Medical School includes approximately 200 Hungarian students per academic year. Student numbers tend to be higher in the lower years and decrease in the upper years due to academic attrition.

### 2.2. Study Design

This serial cross-sectional questionnaire-based study assessed the mental health of medical students from their first to fourth academic years at the University of Pécs Medical School between 2016 and 2019. In this study, ‘original-entry cohort’ refers to students progressing along the standard curriculum without prior academic delay, while ‘repeaters’ refers to students who repeated one or more academic years. Data were collected at five time points: twice during the first year (fall and spring semesters) and once each in the fall semesters of the second, third, and fourth years. Two measurements were taken during the first academic year (fall and spring semesters) to capture changes related to the first exam period, which is known to significantly influence student well-being. To preserve anonymity during the first data collection, students were asked to create a personal code without any identifying information and to use it in subsequent surveys. Despite this, reliable identification of participants across waves was often not possible. Therefore, each wave was treated as an independent cross-sectional study, and aggregated data were analyzed accordingly.

Students were included in a cohort if they were enrolled in a compulsory course scheduled for that year in the standard curriculum. Courses with mandatory seminar attendance were selected, and instructors allocated time during class for questionnaire completion. Students absent or who declined participation were excluded. Participation was voluntary, anonymous, and completed using paper-based questionnaires.

Students were eligible for inclusion if they were enrolled in the General Medicine program, attended the designated seminar or practical session during data collection, and were taking the selected course for the first time in their first academic year (i.e., had established student status in the year of data collection). In the first academic year, all responding students progressed according to the standard curriculum; therefore, there were no repeaters, and all were members of the original-entry cohort. Students were defined as repeaters if they began medical studies before 2016, as determined by a dedicated question in the questionnaire. From the second to the fourth academic years, repeater students’ responses were analyzed separately from those of the original-entry cohort, given the possibly elevated risk of mental health concerns in this student group [28,29].

Table 1 shows the baseline characteristics of the participating students who completed the questionnaire in the given year.

### 2.3. Data Collection

Data collection was conducted using questionnaires, which consisted of three sections:

1. **Demographic data:** Items included age, gender, and the year in which participants’ medical studies were initiated. The mean age of respondents increased from 19.5 to 23.3 years over the four-year study period. Female students consistently comprised the majority, ranging from 58.7% to 60.4%. The proportion of students following the standard curriculum (i.e., the original-entry cohort) declined steadily from 100% in the first year to 56% by the fourth year (Table 1).
2. **Health-related lifestyle factors:**
  - Living arrangements: “Who do you live with during the academic term?” with options including alone, with parents, in a dormitory with others, in a shared rented apartment, or other.
  - Financial situation: “How would you rate your financial situation?” (very good, good, adequate, poor, very poor). For analysis, the categories very good, good, and adequate were grouped separately from the poor and very poor categories.
  - Physical activity: “In an average week, how many times do you engage in physical activity (sports or physical work) lasting more than 20 min, during which your breathing and heart rate increase?” (never, 1–2 times, 3–4 times, ≥5 times), based on Craig et al. (2003) [30]. For analysis, the response options ‘never’ and ‘1–2 times per week’ were combined to form a single category.

It is important to note that at our institution, students are required to complete a compulsory, ungraded physical education course during the first four semesters, intended as a preventive health measure. This requirement should be considered when interpreting temporal changes in physical activity and related outcomes.

3. **Self-rated health:** Assessed using the European Health Interview Survey (EHIS) item: “How is your health in general?” (very good, good, fair, bad, very bad). For statistical analysis, responses were dichotomized as “very good/good” versus all others, following Kjeldsberg et al. (2022) [26,31].
4. **Depressive symptoms:** Measured using the Beck Depression Inventory (BDI; Beck et al., 1988 [32]; Hungarian version: Perczel-Forintos, 2012 [33]), which assesses symptoms over the past week on a 0–3 scale. Total scores were categorized as minimal (0–9), mild (10–18), moderate (19–29), or severe (30–63). Scores ≥10 were considered indicative of clinically relevant depressive symptoms. As a reference population, data from the Hungarostudy 2013 survey were used (Susánszky & Székely, 2013), selecting a subsample matched by age group [33,34]. In our study, the BDI exhibited good internal consistency, with Cronbach’s α values of 0.769 in Wave 1, 0.818 in Wave 2, 0.880 in Wave 3, 0.905 in Wave 4, and 0.924 in Wave 5.

### 2.4. Statistical Analysis

SPSS version 26.0 was used for statistical analysis. Comparisons were conducted using univariate and multivariate logistic regression, the Mann–Whitney U test, correlation analysis (Spearman rank correlation), and stratified analyses. For descriptive statistics, we calculated the mean, standard deviation, and median values. A *p*-value of <0.05 and a 95% confidence interval were used to determine statistical significance. Normality of the data was assessed using the Shapiro–Wilk test, which indicated non-normal distributions; consequently, non-parametric statistical methods were applied. Two participants in the first wave were excluded using listwise deletion due to extensive missing data. For the remaining participants, item-level missing data (<20% per questionnaire) were imputed using the mode for BDI items. No extreme outliers were removed. Logistic regression with a dichotomous BDI cut-off (≥10) was chosen because most predictors were categorical; this approach aligns with previous studies, and it avoids assumptions of normality. The backward conditional method ensured adjustment for confounders while retaining only significant associations. Multivariable logistic regression models were run separately for the original-entry and repeater cohorts, including all relevant variables (age, financial status, living arrangement, and physical activity). Gender was excluded based on prior analyses. Model assumptions were met: the Hosmer–Lemeshow test indicated adequate fit (*p* > 0.05), no influential outliers were identified (standardized residuals, Cook’s distance), continuous variables were linear in the logit (Box–Tidwell test), and there was no multicollinearity (VIF = 1.012–1.179) for either BDI or SRH. Residual autocorrelation was acceptable (Durbin–Watson = 1.705–2.435).

## 3. Results

Given that repeater students formed a heterogeneous subgroup across the data collection waves, temporal analyses were conducted only for students who began their studies in 2016 and followed the standard curriculum (original-entry cohort). To ensure clarity, the two subgroups were analyzed separately, and the presentation of results begins with the original-entry cohort of medical students.

### 3.1. Temporal Changes of Health-Related Lifestyle Factors and Self-Rated Health in the Original-Entry Cohort

During the first academic year (first and second data collection points), most students reported living in dormitories (40.5%, 39.1%), while from the second year onward, shared rental accommodations became most common (35.5%, 34.2%, and 34.4%) (Supplementary Figure S1A).

Only a small proportion of students rated their financial situation as “poor” or “very poor,” and the proportion reporting “good” financial status increased over time, while those selecting “adequate” decreased (Supplementary Figure S1B).

**Figure 1:**
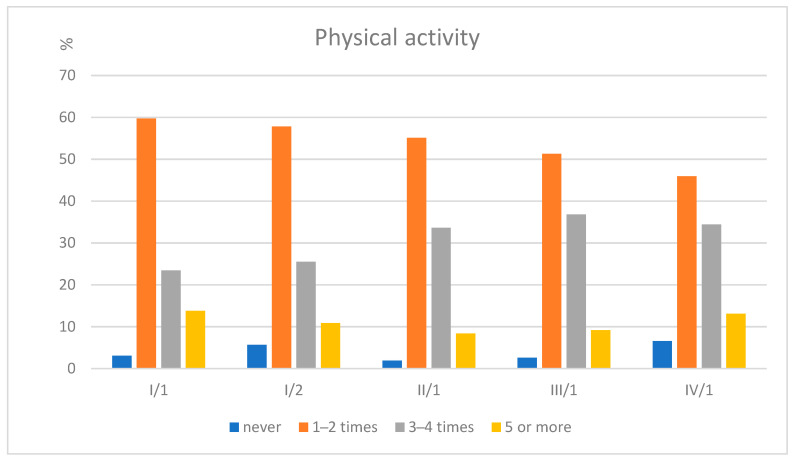
Frequency of self-reported physical activity among medical students from the original-entry cohort.

Most students reported engaging in physical activity multiple times per week. Those reporting no physical activity remained consistently low (<7%), while the proportion exercising 3–4 times weekly increased until the third year and then remained stable (Figure 1).

**Figure 2:**
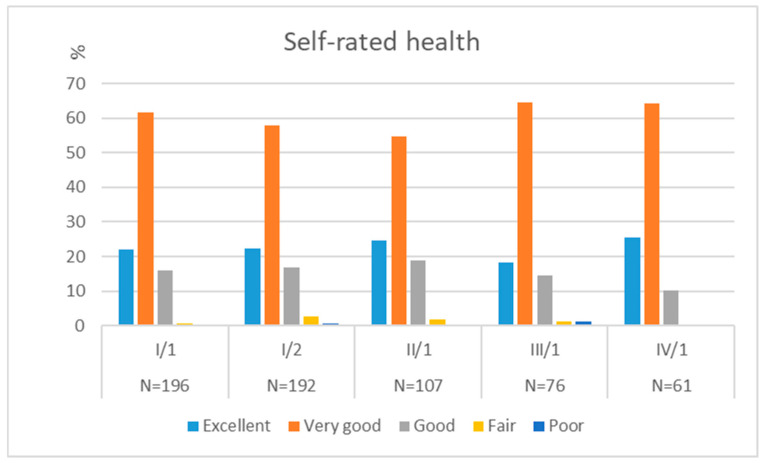
Self-rated health among medical students in the original-entry cohort.

Throughout the four-year study, the majority rated their health as “very good” or “good” (79.2–89.8%), with smaller proportions reporting “satisfactory,” “bad,” or “very bad” (10.2–20.8%). At the first data collection, 83.6% rated their health as very good or good, 15.8% as satisfactory, and 0.5% as bad or very bad. This proportion declined to 79.2% in the second year and increased to 89.8% by the fourth year, the highest observed during the study (Figure 2).

However, no statistically significant differences in self-rated health were observed among students from our original-entry cohort across the data collection points when compared to baseline [H(4) = 2.252; *p* = 0.689].

For comparison, data from the European Health Interview Survey (EHIS, 2014) showed that, within approximately the same age group (15–29 years), 91% rated their health as very good or good, around 8% as satisfactory, and approximately 1% as poor or very poor [35].

### 3.2. Temporal Changes in the BDI Results in the Original-Entry Cohort

**Figure 3:**
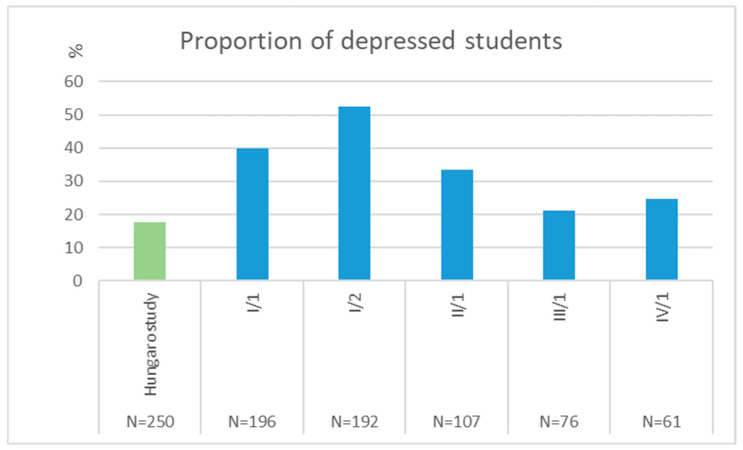
Proportion of medical students exhibiting clinically significant depressive symptoms (BDI scores ≥ 10) in the original-entry cohort.

Among first-year students, nearly 40% exhibited mild or moderate depressive symptoms, increasing to 52.6% by the second semester and then declining to 33.6% in the fourth year and 24.6% by the final year (Figure 3).

Compared with an age-matched general population sample from the Hungarostudy (18–25 years), medical students were significantly more likely to report depressive symptoms during the first three waves of data collection (I/1, *p* < 0.001; I/2, *p* < 0.001; II/1, *p* = 0.001), while the prevalence in waves III/1 (*p* = 0.497) and IV/1 (*p* = 0.214) did not differ significantly from the general population (Figure 3).

Using the first collection as baseline, students were 1.679 times more likely to report depressive symptoms at the beginning of the second semester (OR = 1.679; CI: 1.123–2.511). By the third and fourth years, the odds were approximately half of baseline (Year 3, Semester 1: OR = 0.403; Year 4, Semester 1: OR = 0.493) (Table 2).

Compared to the Hungarostudy general (reference) population, the risk was slightly more than threefold at baseline (OR = 3.095; CI: 2.007–4.773), over fivefold by the second semester (OR = 5.196; CI: 3.375–8.000), and remained elevated in the second year (OR = 2.374; CI: 1.416–3.979) (Table 2).

### 3.3. Factors Influencing Depressive Symptoms and Self-Rated Health in the Original-Entry Cohort

To examine associations between self-reported demographic factors (age, gender) and health-related lifestyle factors (living arrangements, physical activity, financial situation) with both depressive symptoms and self-rated health, correlation analyses were conducted for each of the five data collection waves (see Supplementary Table S1).

Financial difficulties were consistently linked to higher depressive symptoms and poorer self-rated health across waves. More frequent physical activity was associated with better financial situation, lower depressive symptoms in wave I/2, and higher self-rated health in waves I/1, I/2, and II/1. Age showed a weak inverse correlation with self-rated health only in the first wave, and living arrangements were weakly associated with depressive symptoms. Gender was not significantly associated with any of the outcomes. Across all waves, depressive symptoms and self-rated health were moderately inversely correlated.

Financial difficulties were a strong predictor of depressive symptoms. The odds were doubled by the second semester of the first year (OR = 2.064, *p* = 0.003), quadrupled at the start of the second year (OR = 4.127, *p* < 0.001), and remained elevated in the final year (OR = 2.641, *p* = 0.045 (Supplementary Table S2).

Frequent physical activity (OR = 0.639, *p* = 0.033) and living alone (OR = 0.407, *p* = 0.038) reduced depressive symptom likelihood in the second semester of the first year, acting as protective factors. Among third-year students, older age was associated with lower odds of depressive symptoms (OR = 0.444, *p* = 0.045) (Supplementary Table S2).

Older age was associated with poorer self-rated health in the first semester (OR = 0.734, *p* = 0.039). Financial difficulties also remained a strong predictor of poorer self-rated health, with affected students 2–3 times more likely to report unfavorable health (OR range = 0.267–0.441, *p* < 0.047) (Supplementary Table S3).

Physical activity showed a protective effect during the second semester (OR = 2.931, *p* = 0.001), and living with others increased the likelihood of better self-rated health more than threefold (OR = 3.017–3.320, *p* < 0.033) (Supplementary Table S3).

Stratified analysis revealed that physical activity modified the association between financial difficulty and depressive symptoms: financial hardship increased depressive symptom risk 3.5-fold among frequently active students vs. less active students (OR1 = 3.472, CI: 1.184–10.179; OR2 = 7.429, CI: 1.722–32.047).

For self-rated health, effect modification was observed: the association between poor financial status and worse health was significant in both more and less active students, with physical activity slightly reducing the negative effect (OR1 = 4.706, CI: 1.397–11.666; OR2 = 4.037, CI: 1.014–21.831).

### 3.4. Effect of Year Repetition on Depressive Symptoms and Self-Rated Health Among Repeater Students

**Figure 4:**
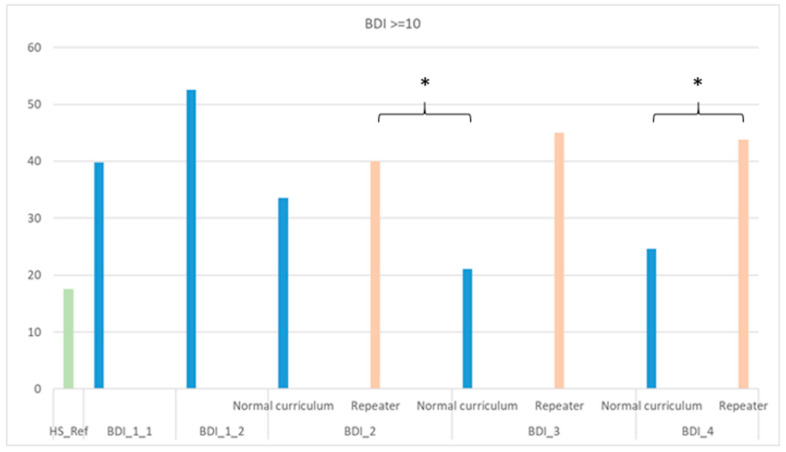
Proportion of repeater students and medical students in the original-entry cohort exhibiting clinically significant depressive symptoms (BDI scores ≥ 10) across academic years. *: *p* < 0.05.

Among second-year repeaters, 40% had mild to moderate depressive symptoms. This rose to 45% in the third year and remained 44% in the fourth year (Figure 4).

Repeaters showed higher odds of depressive symptoms compared to both the general (reference) population and original-entry students across all years (Figure 4, Table 3). Furthermore, as shown in Table 3, when the students’ data are analyzed in aggregate—without treating repeaters and the original-entry cohort as separate subgroups—the estimated risk of depressive symptoms appears substantially lower. Specifically, in the third year, the “Altogether” analysis yields OR = 2.165 *, compared with OR = 3.831 ** among repeaters; likewise, in the fourth year, the corresponding values are OR = 2.309 * for the overall sample and OR = 3.641 ** for repeaters.

Direct comparisons between repeaters and students from the original-entry cohort revealed statistically significant differences in the third and fourth years, with repeaters showing substantially higher odds of depressive symptoms (3rd year: OR = 3.068; CI: 1.449–6.497; 4th year: OR = 2.385; CI: 1.055–5.390).

**Figure 5:**
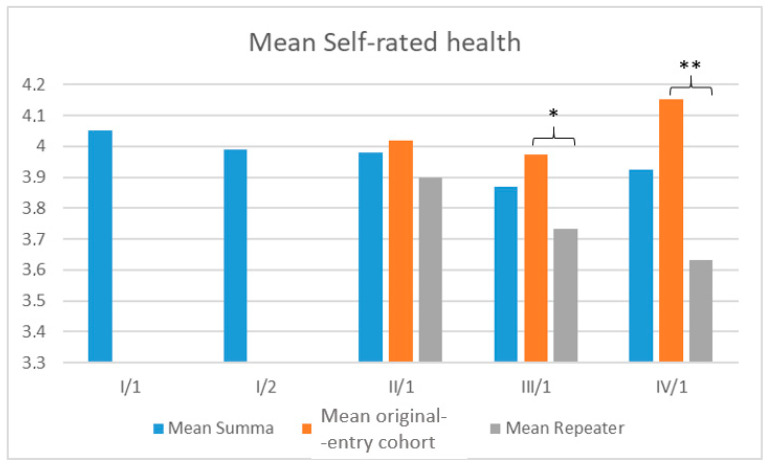
Comparison of self-rated health scores between repeater students and students from the original-entry cohort across academic years. *: *p* < 0.05, ** *p* < 0.001.

Mean self-rated health diverged from the second year, with significant differences in the third (*p* = 0.028) and fourth years (*p* < 0.001), but not in the second year (*p* = 0.471) (Figure 5).

Multivariate analyses within repeaters showed financial difficulties predicted depressive symptoms in the fourth year (OR = 0.187, CI: 0.024). For poor self-rated health, financial hardship was a significant predictor in both the third and fourth years (III/1 OR = 4.044, *p* = 0.014; IV/1 OR = 5.359, *p* = 0.019). No protective factors were identified among repeaters.

## 4. Discussion

To our knowledge, this is one of the few international studies to stratify medical students by repeater status and to monitor changes in their mental health using a repeated cross-sectional design. Our investigation found that physical activity was associated with a protective effect only in the first academic year. Financial difficulties were associated with increased risk throughout almost all of the four years for depressive symptoms and poor SRH among medical students from the original-entry cohort. We found that being a repeater was associated with an over threefold increased odds of having depressive symptoms compared to the general reference population, independent of age, gender, or financial status. We also showed that SRH scores between repeaters and students from our original-entry cohort began to diverge by the second year. Statistically significant differences emerged by the third year and became more pronounced in the fourth.

A longitudinal study in Portugal reported that 12.7% to 21.5% of medical students experienced clinical depression between years 1 and 4, while a study in India found a much higher overall proportion of 64% [22,23]. A large meta-analysis estimated the pooled prevalence of depression or depressive symptoms among medical students at 27.2% [12]. Our findings indicate a somewhat higher proportion than the pooled estimate, with nearly 40% of first-year students from the original-entry cohort exhibiting mild to moderate depressive symptoms. This proportion declined to 25% by the fourth year, with the odds of depressive symptoms in the third and fourth years approximately half those observed in the first year. The BDI cut-off for depressive symptoms applied in our study may have contributed to the higher proportions observed compared to some other reports [12,36,37]. Nevertheless, most studies on medical students’ mental health—including the Hungarostudy—have used the same 10-point threshold for depressive symptoms [1,38,39].

In the first two academic years, medical students in our original-entry cohort had significantly higher odds of depressive symptoms compared with the reference Hungarian population, with first- and second-year students showing 2.4- to 5.2-fold higher odds. This elevated burden of depressive symptoms among medical students, relative to the general population and non-medical peers, has been consistently documented in earlier work [1]. By the 3rd and 4th years, the proportion in our sample did not differ significantly from that reported in the Hungarostudy, possibly reflecting reduced stress through better adaptation and increased self-confidence [34]. However, this improvement was confined to students in our original-entry cohort; among repeaters, the proportion of students with depressive symptoms remained significantly and consistently higher across all academic years compared with the Hungarostudy.

Similar trends investigating medical students have been observed in other countries: in a German study, Pukas et al. reported a steep increase in depressive symptom scores from the 1st to the 4th semester (*p* = 0.011), followed by relative stagnation between the 4th and 5th semesters (*p* = 0.679), and then a further significant rise by the 9th/10th semester (both *p* < 0.001) [40]. This pattern suggests that while early academic years may be marked by a sharp escalation in the scores of depressive symptoms, later phases can show periods of stabilization, potentially before another increase as clinical and professional pressures mount [40,41].

In previous studies, self-rated health among medical students showed variable trends over time. In one German cohort, the proportion of students reporting good health declined from 93% to 76% during the first year of study, whereas a five-year longitudinal study found consistently high rates (95.8–98.6%) of good to excellent general health, with only minor year-to-year fluctuations [42,43]. In our original-entry cohort, although no statistically significant change in self-rated health was observed overall, we also noted a slight decrease in self-reported health by the third year, followed by a modest increase from the first to the fourth year of data collection.

Consistent with evidence that regular physical activity is associated with lower depressive symptoms and anxiety, our original-entry cohort showed that higher frequency of exercise was associated with reduced depressive symptoms and better SRH during the early academic years [1,17,44,45]. Most medical students in our original-entry cohort reported engaging in physical activity several times per week, with time series analysis indicating an increase in those exercising 3–4 times weekly. By the second semester of the first year, both frequent physical activity and living alone were associated with a reduced likelihood of depressive symptoms. Frequent physical activity was also linked to a threefold increase in positive self-rated health, while students living with others were more than three times as likely to report better health than those living alone.

An earlier study at the Faculty of Medicine of Semmelweis University similarly found that vigorous exercise was associated with better mental health, and population-based data indicate that individuals not engaging in regular physical activity are twice as likely to exhibit symptoms of depression (PR = 2.1) and anxiety (PR = 2.5) compared with those who are physically active [1,45]. Despite a significant proportion of students in the original-entry cohort still not meeting the WHO recommendations for physical activity, at least their physical activity did not decline following the end of compulsory physical education in the 5th semester [46]. This can therefore be regarded as a positive trend.

Our findings further suggest that physical activity may be associated with the mitigation of the adverse mental health effects of financial difficulties, a factor of particular relevance given evidence from a systematic review showing that higher debt among medical students is linked to poorer mental health and worse academic performance [19]. Additionally, family and relational factors, such as overprotective or “helicopter” parenting, have been associated with higher depressive symptoms and lower self-esteem in university students, highlighting the broader psychosocial determinants of mental health [47,48,49]. In the upper academic years, poorer self-reported mental health was associated with higher levels of physical activity. While causality cannot be inferred, this pattern may indicate increased self-care awareness and recognition of exercise benefits reinforced through curricular and extracurricular experiences.

The year-to-year variation in the protective effect of physical activity in our study can be explained by several factors. In the early years of training, students more frequently participate in structured, often moderate-to-vigorous forms of physical activity, which have been shown to improve mental health [50]. In later years, increasing academic workload and lack of time become major barriers to physical activity [1], while stress and burnout levels typically rise [12]. Self-selection may also occur: those who remain active are more likely to be health-conscious or to have better baseline mental health [51]. It is also important to note that our study measured frequency rather than intensity, although moderate-to-vigorous intensity activity has the greatest beneficial effect on mental health [52]. This measurement difference may contribute to the finding that the protective effect was observed only in the early years.

Repeater students showed significantly higher odds of depressive symptoms compared to both the Hungarostudy reference population and peers from our original-entry cohort. From years two to four, their odds were more than three times higher than the general population, with a progressive increase over time. Without distinguishing repeating students from original-entry cohorts, key associations—such as the increased risk of depressive symptoms relative to the general population (Table 3)—may remain undetected. Our findings highlight the importance of subgroup-specific analyses to guide future research and data collection on medical student mental health.

Our results revealed a divergence in SRH trajectories: while repeaters and original-entry students initially reported comparable self-rated health, by the third academic year, repeaters showed markedly poorer SRH, with the gap widening further in the fourth year. This trend may reflect the cumulative effects of stress, stigma, or social isolation associated with delayed academic progression.

Physical activity, which was associated with a protective effect for students from our original-entry cohort, did not show a protective effect among repeaters; in fact, no protective factors were identified for repeater students within the examined parameters. However, financial difficulties emerged as significant risk factors for poor self-rated health in the third and fourth years, as well as for depressive symptoms among repeaters [53,54]. Repeater students face numerous challenges, including frequent feelings of shame, fear of peer judgment, diminished self-esteem, and concerns about the financial consequences of repeating an academic year [55]. The association between repeater status and poorer mental health found in our study suggests that mental health problems may contribute to academic difficulties. This aligns with previous research showing that students facing mental health challenges are at greater risk of dropping out of university [56,57,58].

*Implications*

Academic year repetition can result from a combination of academic, personal, and mental health factors, with depressive and anxiety symptoms being consistently associated with increased risk of delayed progression or academic underperformance among medical students [1]. The vulnerabilities of repeater students may be explained by several psychological mechanisms. Prior academic failures can reduce self-efficacy, resulting in greater anxiety and underperformance [59,60]. Feelings of shame associated with failure further undermine adaptive coping by promoting a global negative self-evaluation [61,62]. In addition, the situation of repeater students may intensify negative social comparisons, which can lower self-esteem and increase psychological vulnerability [63]. Our results have important implications for medical education policy and student support services. Recognizing that repeater students are at substantially higher risk of depressive symptoms and worsening self-rated health highlights the need for early, targeted interventions. Medical schools should consider routine mental health screening, peer mentoring, and remedial academic support tailored specifically to those who fall behind. While general wellness programs promoting exercise are likely beneficial, repeaters may require more comprehensive support—including psychosocial counseling, flexible curricula, and peer integration strategies—to address potential emotional challenges associated with academic repetition [64,65,66].

This focus on student mental health is especially critical given that physicians influence both patient outcomes and lifestyle choices by modeling healthy behaviors. Research indicates that doctors’ personal habits—such as exercise and substance use—are linked to their patient counseling practices, with many of these behaviors established prior to medical school [15,20,21,22,23,26]. Therefore, ensuring medical students can effectively recognize and manage their mental health is essential not only for their own well-being but also to support their future role as credible health promoters and role models.

*Limitations*

Several limitations should be noted. Attrition may have introduced self-selection bias, as students who dropped out or did not respond in later years might differ systematically from those who participated consistently, potentially affecting the observed outcomes. The single-institution setting limits generalizability, given potential cultural and curricular differences. Our reliance on self-report measures, including the Beck Depression Inventory and the single-item self-rated health measure, may have led to underestimation or overestimation of depressive symptoms and health status, as responses are subject to individual perception and reporting biases. Data on prior psychiatric history, personality traits, and coping mechanisms were not collected, which could influence outcomes. The observational design precludes causal inference: we can identify associations between depressive symptoms and academic repetition, but cannot determine the direction of effect, and repeater status was not randomly assigned. Additionally, the single-item self-rated health measure may lack sensitivity compared to multi-item instruments.

Another limitation of this study is the approximately three-year gap between the student sample and available national data, which may have affected comparability. No nationally representative survey was conducted in 2016, and the 2020 Hungarostudy data were considered unsuitable as a reference due to the COVID-19 pandemic’s substantial impact on population mental health. Therefore, comparisons of the student sample’s baseline status should be interpreted with caution.

*Future Directions*

Nonetheless, following the same cohort with high retention over four years allowed for a reliable analysis of changes over time. Stratification by repeater status provided a level of detail uncommon in medical education research, particularly in Central Europe. Use of validated measures, including the Beck Depression Inventory and consistent self-rated health scales, enhanced internal validity. Finally, this study provides region-specific data on Hungarian medical students, which may serve as a useful reference for future research in Central and Eastern Europe. Future studies should examine multi-institutional cohorts across the region to confirm the generalizability of these findings and further explore the longitudinal impact of academic repetition on student mental health, including potential interventions to mitigate adverse outcomes.

## 5. Conclusions

In our repeated cross-sectional study of Hungarian medical students, we found that depressive symptoms were substantially more prevalent than in an age-matched general population, particularly during the early years of training. Students in the original-entry cohort, i.e., those who began their studies in 2016 and followed the standard curriculum, generally showed improvement over time; in contrast, those repeating academic years experienced more than triple the odds of depressive symptoms and a decline in self-rated health by later years. Highlighting this subgroup of repeaters is a key contribution of the study, as it identifies students at particular risk who may benefit from targeted support or interventions.

Financial hardship emerged as a strong and consistent risk factor for both depressive symptoms and poorer self-rated health, emphasizing the critical need for early detection and support for economically disadvantaged students. Frequent physical activity was associated with better mental health, particularly in the initial years of medical training, suggesting that promoting healthy lifestyle behaviors may help mitigate mental health risks.

Supporting medical students’ mental well-being is important not only for their personal health but also to enhance their future effectiveness and quality of care as healthcare professionals. Therefore, our findings provide valuable insights to inform the development of preventative programs and support services within medical education institutions.

## Figures and Tables

**Table 1:** Baseline characteristics of participating students at the University of Pécs Medical School (UP MS) in academic years 1–4.

Data Collection (Year/Semester)	Age (Mean, SD)	Gender (%)		Students Ratio (%)		Respondents (n)	All Students per Year (n)	Response Rate (%)
		**Male**	**Female**	**Original-Entry** **Cohort**	**Repeater**			
**I/1.**	19.48 (1.28)	39.80	59.70	100.00	0.00	196	203	96.6%
**I/2.**	19.81 (1.41)	38.50	60.40	100.00	0.00	192	193	99.5%
**II/1.**	20.70 (1.34)	36.30	59.90	68.20	31.80	157	182	86.3%
**III/1.**	22.35 (1.73)	39.70	59.60	55.90	44.10	136	147	92.5%
**IV/1.**	23.29 (1.81)	38.50	58.70	56.00	44.00	109	155	70.3%

**Table 2:** Probability of depressive symptoms relative to the Hungarostudy (reference population) and baseline assessment (1st year/1st semester) results. OR: odds ratio, CI: confidence interval, *: *p* < 0.05; **: *p* < 0.001.

Hungarostudy Versus…					I/1. Versus…				
	OR	CI	Χ^2^	*p*		OR	CI	Χ^2^	*p*
**I/1.**	3.095 **	2.007–4.773	27.238	<0.001	-	-	-	-	-
**I/2.**	5.196 **	3.375–8.000	60.364	<0.001	**I/2.**	1.679 *	1.123–2.511	6.403	0.011
**II/1.**	2.374 **	1.416–3.979	11.094	<0.001	**II/1.**	0.767	0.469–1.255	1.116	0.291
**III/1.**	1.248	0.658–2.369	0.463	0.496	**III/1.**	0.403 *	0.217–0.751	8.507	0.004
**IV/1.**	1.527	0.783–2.976	1.559	0.212	**IV/1.**	0.493 *	0.258–0.944	4.658	0.031

**Table 3:** Odds ratios for depressive symptoms among repeater students vs. students in the original-entry cohort and compared to the Hungarostudy population OR: odds ratio, *p*: significance. *: *p* < 0.05, ** *p* < 0.001.

		Altogether			Original-Entry Cohort			Repeater		
Reference	Data Collection	OR (CI)	Χ^2^	*p*	OR (CI)	Χ^2^	*p*	OR (CI)	Χ^2^	*p*
Hungarostudy	I/1.	3.095 ** (2.007–4.773)	27.238	<0.001	3.095 * (2.007–4.773)	27.238	<0.001	no repeater participated		
	I/2.	5.196 ** (3.375–8.000)	60.364	<0.001	5.196 * (3.375–8.000)	60.364	<0.001	no repeater participated		
	II/1.	2.596 ** (1.637–4.116)	16.988	<0.001	2.374 * (1.416–3.979)	11.094	<0.001	3.121 ** (1.625–5.995)	12.458	<0.001
	III/1.	2.165 * (1.331–3.521)	9.913	0.002	1.248 (0.658–2.369)	0.463	0.496	3.831 ** (2.094–7.007)	20.573	<0.001
	IV/1.	2.309 * (1.379–3.864)	10.432	0.001	1.527 (0.783–2.976)	1.559	0.212	3.641 ** (1.888–7.022)	16.146	<0.001

## Data Availability

The original contributions presented in this study are included in the article/Supplementary Material. Further inquiries can be directed to the corresponding author.

## Supplementary Materials

The following supporting information can be downloaded at: https://www.mdpi.com/article/10.3390/jcm14238447/s1, Supplementary Figure S1: Health-related lifestyle factors: living arrangements (A) and financial situation (B) among students from the original-entry cohort. Supplementary Table S1: Correlation analysis of demographic and health-related lifestyle factors associated with depressive symptoms and self-rated health in students from our original-entry cohort. Supplementary Table S2: Significant predictive factors of depressive symptoms across data collection waves among students in the original-entry cohort. Supplementary Table S3: Significant predictive factors of self-rated health across data collection waves among students in the original-entry cohort.

## Abbreviations

BDI	Beck Depression Inventory
CI	95% Confidence Interval
EHIS	European Health Interview Survey
GHQ	General Health Questionnaire
OR	Odds Ratio
SRH	Self-Rated Health
