# Supplementary material for: Mental Health Trajectories in Medical Students: The Impact of Academic Repetition on Depressive Symptoms and Self-Rated Health
Source: J Clin Med. 2025 Nov 28;14(23):8447. doi: 10.3390/jcm14238447 (PMC12692891; doi:10.3390/jcm14238447)
Supplement: Supplementary file 1 [file jcm-14-08447-s001.zip › jcm-3967270-supplementary.pdf]

**Supplementary Materials:**

***Supplementary Figure S1: Health-related lifestyle factors among medical students from the original-entry cohort.***

*Panel A shows the distribution of living arrangements reported by students during the academic term, including categories such as living alone, with parents, in dormitory with others, or shared rented apartments.*

*Panel B presents students' self-rated financial situation, divided into categories: very good, good, adequate, poor, and very poor.*

*Data are shown as percentages of respondents in each category across the five data collection time points.*

***A)***

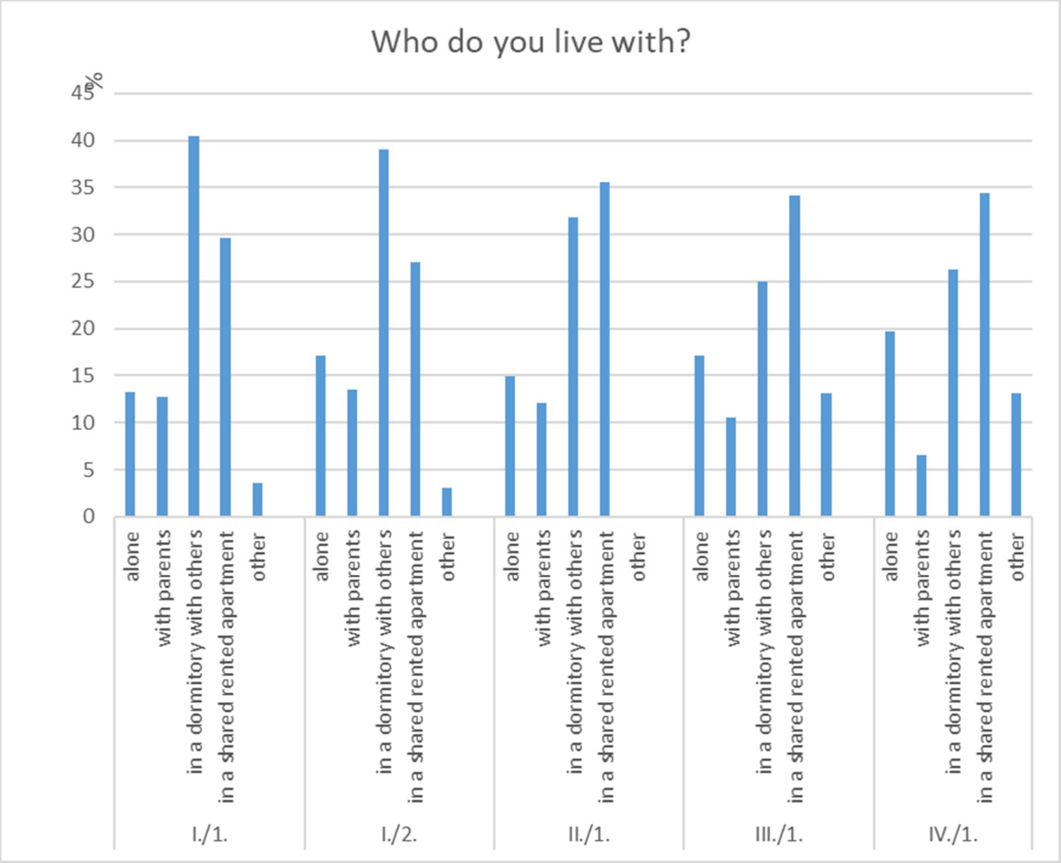


*B)*

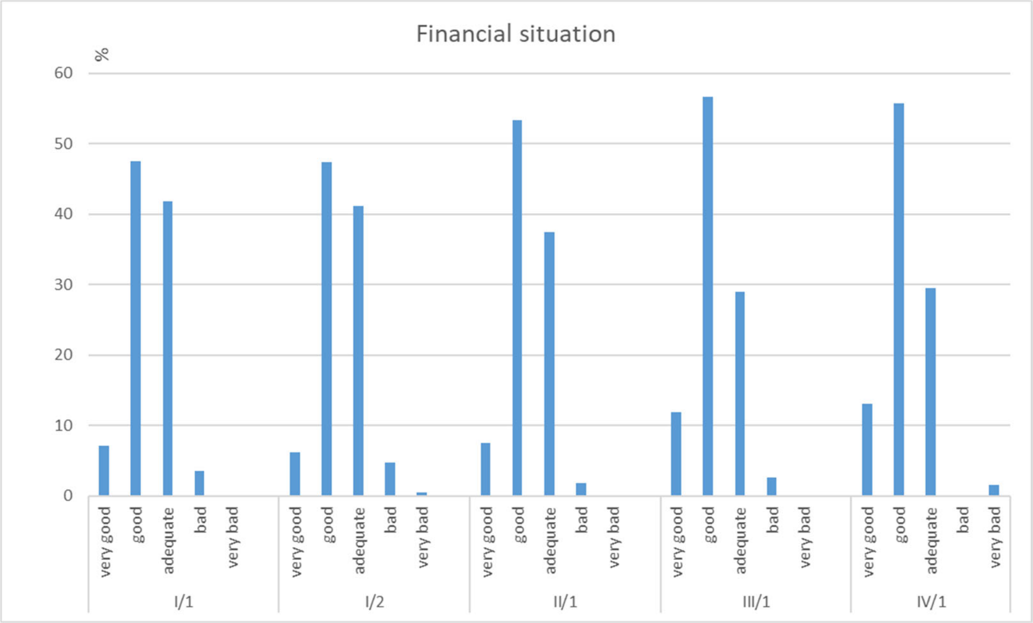


***Supplementary Table S1:*** Spearman's correlation coefficients (r) and p-values (p) for associations between demographic variables (age, gender), health-related lifestyle factors (physical activity frequency, financial situation, living arrangements), depressive symptoms (Beck Depression Inventory scores), and self-rated health across five data collection waves in the original-entry cohort. Significant correlations are indicated as the following: *: $p<0.05$, **$p<0.001$ .

| Wave | | | Age | Gender | Financial situation | Living | Physical | BDI | SRH |
|---|---|---|---|---|---|---|---|---|---|
| I./1 | Age | r | 1.000 | -0.042 | 0.128 | 0.,034 | -0.029 | 0.136 | -0.174* |
| | | p | . | 0.562 | 0.073 | 0.637 | 0.,683 | 0.058 | 0.015 |
| | | N | 196 | 195 | 196 | 195 | 196 | 196 | 196 |
| | Gender | r | -0.042 | 1.000 | -0.089 | -0.042 | -0.023 | 0.005 | 0.032 |
| | | p | 0.562 | . | 0.214 | 0.564 | 0.751 | 0.940 | 0.654 |
| | | N | 195 | 195 | 195 | 194 | 195 | 195 | 195 |
| | Financial situation | r | 0.128 | -0.089 | 1.000 | 0.188** | -0.210** | 0.041 | -0.289** |
| | | p | 0.073 | 0.214 | . | 0.008 | 0.003 | 0.565 | <0.001 |
| | | N | 196 | 195 | 196 | 195 | 196 | 196 | 196 |
| | Living | r | 0.034 | -0.042 | 0.188** | 1.000 | -0.067 | -0.063 | -0.078 |
| | | p | 0.637 | 0.564 | 0.008 | . | 0.354 | 0.379 | 0.279 |
| | | N | 195 | 194 | 195 | 195 | 195 | 195 | 195 |
| | Physical | r | -0.029 | -0.023 | -0.210** | -0.067 | 1.000 | -0.100 | 0.249** |
| | | p | 0.683 | 0.751 | 0.003 | 0.354 | . | 0.162 | <0.001 |
| | | N | 196 | 195 | 196 | 195 | 196 | 196 | 196 |
| | BDI | r | 0.136 | 0.005 | 0.041 | -0.063 | -0.100 | 1.000 | -0.334** |
| | | p | 0.058 | 0.940 | 0.565 | 0.379 | 0.162 | . | <0.001 |
| | | N | 196 | 195 | 196 | 195 | 196 | 196 | 196 |
| | SRH | r | -0.174* | 0.032 | -0.289** | -0.078 | 0.249** | -0.334** | 1.000 |
| | | p | 0.015 | 0.654 | <0.001 | 0.279 | <0.001 | <0.001 | . |

| | | | | | | | | | |
|---|---|---|---|---|---|---|---|---|---|
| | | N | 196 | 195 | 196 | 195 | 196 | 196 | 196 |
| I./2 | Age | r | 1.000 | -0.067 | 0.106 | -0.036 | 0.068 | 0.050 | -0.004 |
| | | p | . | 0.355 | 0.144 | 0.624 | 0.346 | 0.492 | 0.955 |
| | | N | 192 | 190 | 192 | 192 | 192 | 192 | 192 |
| | Gender | r | -0.067 | 1.000 | -0.016 | 0.034 | -0.054 | -0.087 | 0.023 |
| | | p | 0.355 | . | 0.828 | 0.641 | 0.456 | 0.233 | 0.758 |
| | | N | 190 | 190 | 190 | 190 | 190 | 190 | 190 |
| | Financial situation | r | 0.106 | -0.016 | 1.000 | 0.161* | -0.202* | 0.227* | -0.327** |
| | | p | 0.144 | 0.828 | . | 0.026 | 0.005 | 0.002 | <0.001 |
| | | N | 192 | 190 | 192 | 192 | 192 | 192 | 192 |
| | Living | r | -0.036 | 0.034 | 0.161* | 1.000 | -0.032 | -0.178* | -0.024 |
| | | p | 0.624 | 0.641 | 0.026 | . | 0.662 | 0.013 | 0.743 |
| | | N | 192 | 190 | 192 | 192 | 192 | 192 | 192 |
| | Physical | r | 0.068 | -0.054 | -0.202** | -0.032 | 1.000 | -0.233** | 0.395** |
| | | p | 0.346 | 0.456 | 0.005 | 0.662 | . | 0.001 | <0.001 |
| | | N | 192 | 190 | 192 | 192 | 192 | 192 | 192 |
| | BDI | r | 0.050 | -0.087 | 0.227* | -0.178* | -0.233** | 1.000 | -0.366** |
| | | p | 0.492 | 0.233 | 0.002 | 0.013 | 0.001 | . | <0.001 |
| | | N | 192 | 190 | 192 | 192 | 192 | 192 | 192 |
| | SRH | r | -0.004 | 0.023 | -0.327** | -0.024 | 0.395** | -0.366** | 1.000 |
| | | p | 0.955 | 0.758 | <0.001 | 0.743 | <0.001 | <0.001 | . |

| | | | | | | | | | |
|---|---|---|---|---|---|---|---|---|---|
| | | N | 192 | 190 | 192 | 192 | 192 | 192 | 192 |
| II./1 | Age | r | 1<0.001 | -0.016 | 0.207* | 0.065 | 0.040 | 0.111 | -0.138 |
| | | p | . | 0.872 | 0.035 | 0.528 | 0.690 | 0.264 | 0.164 |
| | | N | 104 | 103 | 104 | 98 | 103 | 104 | 103 |
| | Gender | r | -0.016 | 1.000 | 0.001 | 0.110 | -0.046 | 0.076 | -0.041 |
| | | p | 0.872 | . | 0.991 | 0.285 | 0.643 | 0.446 | 0.679 |
| | | N | 103 | 103 | 103 | 97 | 102 | 103 | 102 |
| | Financial situation | r | 0.207* | 0.001 | 1.000 | 0.184 | -0.103 | 0.435** | -0.392** |
| | | p | 0.035 | 0.991 | . | 0.066 | 0.292 | <0.001 | <0.001 |
| | | N | 104 | 103 | 107 | 101 | 106 | 107 | 106 |
| | Living | r | 0.065 | 0.110 | 0.184 | 1.000 | -0.073 | 0.146 | -0.187 |
| | | p | 0.528 | 0.285 | 0.066 | . | 0.469 | 0.144 | 0.063 |
| | | N | 98 | 97 | 101 | 101 | 100 | 101 | 100 |
| | Physical | r | 0.040 | -0.046 | -0.103 | -0.073 | 1.000 | -0.174 | 0.315** |
| | | p | 0.690 | 0.643 | 0.292 | 0.469 | . | 0.075 | 0.001 |
| | | N | 103 | 102 | 106 | 100 | 106 | 106 | 105 |
| | BDI | r | 0.111 | 0.076 | 0.435** | 0.146 | -0.174 | 1.000 | -0.532** |
| | | p | 0.264 | 0.446 | <0.001 | 0.144 | 0.075 | . | <0.001 |
| | | N | 104 | 103 | 107 | 101 | 106 | 107 | 106 |
| | SRH | r | -0.138 | -0.041 | -0.392** | -0.187 | 0.315** | -0.532** | 1.000 |
| | | p | 0.164 | 0.679 | <0.001 | 0.063 | 0.001 | <0.001 | . |

| | | | | | | | | | |
|---|---|---|---|---|---|---|---|---|---|
| | | N | 103 | 102 | 106 | 100 | 105 | 106 | 106 |
| III./1 | Age | r | 1.000 | -0.092 | 0.135 | -0.116 | 0.115 | -0.113 | -0.053 |
| | | p | . | 0.431 | 0.245 | 0.318 | 0.322 | 0.330 | 0.652 |
| | | N | 76 | 76 | 76 | 76 | 76 | 76 | 76 |
| | Gender | r | -0.092 | 1.000 | 0.077 | -0.046 | -0.028 | 0.138 | 0.053 |
| | | p | 0.431 | . | 0.510 | 0.691 | 0.813 | 0.233 | 0.647 |
| | | N | 76 | 76 | 76 | 76 | 76 | 76 | 76 |
| | Financial situation | r | 0.135 | 0.077 | 1.000 | 0.218 | -0.098 | 0.256* | -0.452** |
| | | p | 0.245 | 0.510 | . | 0.058 | 0.400 | 0.025 | <0.001 |
| | | N | 76 | 76 | 76 | 76 | 76 | 76 | 76 |
| | Living | r | -0.116 | -0.046 | 0.218 | 1.000 | -0.173 | 0.014 | -0.078 |
| | | p | 0.318 | 0.691 | 0.058 | . | 0.134 | 0.906 | 0.504 |
| | | N | 76 | 76 | 76 | 76 | 76 | 76 | 76 |
| | Physical | r | 0.115 | -0.028 | -0.098 | -0.173 | 1.000 | -0.018 | 0.187 |
| | | p | 0.322 | 0.813 | 0.400 | 0.134 | . | 0.875 | 0.106 |
| | | N | 76 | 76 | 76 | 76 | 76 | 76 | 76 |
| | BDI | r | -0.113 | 0.138 | 0.256* | 0.014 | -0.018 | 1.000 | -0.502** |
| | | p | 0.330 | 0.233 | 0.025 | 0.906 | 0.875 | . | <0.001 |
| | | N | 76 | 76 | 76 | 76 | 76 | 76 | 76 |
| | SRH | r | -0.053 | 0.053 | -0.452** | -0.078 | 0.187 | -0.502** | 1.000 |
| | | p | 0.652 | 0.647 | <0.001 | 0.504 | 0.106 | <0.001 | . |

| | | | | | | | | | |
|---|---|---|---|---|---|---|---|---|---|
| | | N | 76 | 76 | 76 | 76 | 76 | 76 | 76 |
| IV./1 | Age | r | 1.000 | -0.236 | 0.129 | -0.084 | 0.079 | 0.040 | -0.197 |
| | | p | . | 0.074 | 0.331 | 0.526 | 0.552 | 0.761 | 0.143 |
| | | N | 59 | 58 | 59 | 59 | 59 | 59 | 57 |
| | Gender | r | -0.236 | 1.000 | 0.063 | 0.082 | 0.017 | 0.035 | -0.123 |
| | | p | 0.074 | . | 0.640 | 0.539 | 0.898 | 0.793 | 0.360 |
| | | N | 58 | 58 | 58 | 58 | 58 | 58 | 57 |
| | Financial situation | r | 0.129 | 0.063 | 1.000 | 0.122 | -0.218 | 0.430** | -0.525** |
| | | p | 0.331 | 0.640 | . | 0.347 | 0.091 | 0.001 | <0.001 |
| | | N | 59 | 58 | 61 | 61 | 61 | 61 | 59 |
| | Living | r | -0.084 | 0.082 | 0.122 | 1.000 | -0.137 | 0.162 | -0.113 |
| | | p | 0.526 | 0.539 | 0.347 | . | 0.294 | 0.213 | 0.393 |
| | | N | 59 | 58 | 61 | 61 | 61 | 61 | 59 |
| | Physical | r | 0.079 | 0.017 | -0.218 | -0.137 | 1.000 | -0.133 | 0.142 |
| | | p | 0.552 | 0.898 | 0.091 | 0.294 | . | 0.306 | 0.283 |
| | | N | 59 | 58 | 61 | 61 | 61 | 61 | 59 |
| | BDI | r | 0.040 | 0.035 | 0.430** | 0.162 | -0.133 | 1.000 | -0.504** |
| | | p | 0.761 | 0.793 | 0.001 | 0.213 | 0.306 | . | <0.001 |
| | | N | 59 | 58 | 61 | 61 | 61 | 61 | 59 |
| | SRH | r | -0.197 | -0.123 | -0.525** | -0.113 | 0.142 | -0.504** | 1.000 |

| | | | | | | | | |
|---|---|---|---|---|---|---|---|---|
| | p | 0.143 | 0.360 | <0.001 | 0.393 | 0.283 | <0.001 | . |
| | N | 57 | 57 | 59 | 59 | 59 | 59 | 59 |

**Supplementary Table S2:** Significant predictive factors of depressive symptoms across data collection waves among students in the original-entry cohort.* OR: Odds ratio, p: significance.

| **Data collection→** | **I/1.** | | **I/2.** | | **II/1.** | | **III./1.** | | **IV./1.** | |
|---|---|---|---|---|---|---|---|---|---|---|
| | **N=196** | | **N=192** | | **N=107** | | **N=76** | | **N=61** | |
| **Predictors↓** | **OR** | **p** | **OR** | **p** | **OR** | **p** | **OR** | **p** | **OR** | **p** |
| **Risk factors** | | | | | | | | | | |
| Financial concerns | - | | 2.064 | 0.003 | 4.127 | <0.001 | - | | 2.641 | 0.045 |
| Protective factors | | | | | | | | | | |
| Older age | - | - | | - | - | | 0.444 | 0.039 | - | |
| Physical activity | - | - | 0.639 | 0.033 | - | | - | | - | |
| Living arrangement (living alone) | - | - | 0.407 | 0.038 | - | | - | | - | |

*Note: Only significant results are shown. A backward conditional method was applied in the logistic regression, retaining only significant associations in the stepwise analysis.

**Supplementary Table S3.** : Significant predictive factors of self-rated health across data collection waves among students in the original-entry cohort. OR: Odds ratio, p: significance.

| **Data collection→** | **I/1.** | | **I/2.** | | **II/1.** | | **III./1.** | | **IV./1.** | |
|---|---|---|---|---|---|---|---|---|---|---|
| | **N=196** | | **N=192** | | **N=107** | | **N=76** | | **N=61** | |
| **Predictors↓** | **OR** | **p** | **OR** | **p** | **OR** | **p** | **OR** | **p** | **OR** | **p** |
| **Risk factors** | | | | | | | | | | |
| Financial concerns | 0.286 | 0.000 | 0.424 | 0.006 | 0.441 | 0.047 | 0.267 | 0.012 | - | - |

| | | | | | | | | | | |
|---|---|---|---|---|---|---|---|---|---|---|
| Older age | 0.734 | 0.039 | - | - | - | - | - | - | - | - |
| **Protective factors** | | | | | | | | | | |
| Physical activity | - | - | 2.931 | 0.001 | - | - | - | - | - | - |
| Living arrangement (living with others) | 3.320 | 0.032 | 3.017 | 0.033 | - | - | - | - | - | - |

*Note: Only significant results are shown. A backward conditional method was applied in the logistic regression, retaining only significant associations in the stepwise analysis.
